# Next-generation linkage and association methods applied to hypertension: a multifaceted approach to the analysis of sequence data

**DOI:** 10.1186/1753-6561-8-S1-S111

**Published:** 2014-06-17

**Authors:** William CL Stewart, Yungui Huang, David A Greenberg, Veronica J Vieland

**Affiliations:** 1 Nationwide Children's Hospital, 700 Children's Drive Columbus, Ohio 43205, USA

## Abstract

To realize the full potential of next-generation sequencing, it is important to consider multiple sources of genetic information, including inheritance, association, and bioinformatics. To illustrate the promise of such an approach, we applied our next-generation linkage and association (NGLA) methods to the sequence data of a large 57-member Mexican American family with hypertension. Our results show that *OSBPL10*--a disease susceptibility gene for dyslipidemia--may also influence systolic blood pressure (SBP). In particular, our NGLA dense single-nucleotide polymorphism (SNP) analysis identified a 2.5-megabase (Mb) region that strongly cosegregates with low SBP (maximum posterior probability of linkage [PPL] = 68%). Furthermore, using the posterior probability of linkage disequilibrium (PPLD), we fine-mapped this region and identified 12 SBP-associated variants (PPLD ranging between 4% and 14%) that comprise a rare, 4-site haplotype. This haplotype extends into the candidate gene, *OSBPL10 *(oxysterol-binding protein-like 10). In contrast to our NGLA methods, a commonly used filter-based approach identified 23 variants with little evidence for spatial clustering around any particular gene or region of interest.

## Background

The Genetic Analysis Workshop 18 (GAW18) distributed phenotype, genotype, and sequence data on 20 large Mexican American families. For 772 individuals who were not taking medication for hypertension, SBP was measured repeatedly with a maximum of 4 observations per person. Eighty-four individuals were actively taking hypertension medication, and the phenotypes of 530 individuals were unobserved. For individuals who were not taking medication for hypertension, we used SBP at the first available time point as our quantitative phenotype and regarded treated individuals (ie, those taking hypertension medication) as affected. All analyses were performed on the unadjusted phenotype data without correction for covariates, and, as recommended by the GAW18 Program Committee, we restricted our attention to chromosome 3.

## Methods

We analyzed the phenotypic data under a quantitative trait threshold model using the program KELVIN [1], which integrates out all parameters of the trait model, including the threshold parameter. Therefore, with this model, the SBP values of untreated individuals were analyzed jointly with the affected status of treated individuals. When the quantitative trait threshold model is used in conjunction with a model for the observed genotypes, the probabilities of linkage and linkage disequilibrium (LD) can be computed without fixing the parameters of the trait model. However, if desired, estimates of these parameters (ie, posterior means) are easily obtained as a by-product of the calculations required for the PPL. To mitigate the negative effects of genotyping error, we applied standard quality-control procedures. As a result, SNPs with minor allele frequencies less than 0.01 and SNPs with Hardy-Weinberg equilibrium *p *values less than 0.0001 were removed.

To maximize the chance of detecting linkage, we analyzed all 20 families across a relatively sparse, but highly informative subsample of the 65,519 genome-wide association study (GWAS) SNPs. In particular, the selected subsample contained 719 SNPs with an average heterozygosity of 43% and a maximum pairwise *r*^2 ^between adjacent SNPs of 0.20. The genetic map was obtained from the Rutger's published map [2], and the PPL was computed using KELVIN. The pedigree-based likelihoods for the very large families were computed using a novel hybrid Markov chain Monte Carlo (MCMC) approach. Specifically, the MCMC handled the genotype data [3], while a fast and accurate adaptive quadrature method handled the phenotype data [4]. As a precaution, we also used the program EAGLET [5,6] in conjunction with LM_MARKERS [7] to assess the evidence for linkage. Note that EAGLET generates multiple random, informative subsamples from dense SNP genotype data, and that LM_MARKERS estimates model-based pedigree likelihoods for large families. Therefore, the coordinated use of both programs provides inference about cosegregation that is robust to linkage map construction.

To identify SBP susceptibility genes (ie, to fine-map underneath a linkage peak), we selected the pedigree with the largest PPL on chromosome 3 and applied our NGLA methods to the sequence data of a 2.5 Mb region covering the peak. These methods include (but are not limited to) a bayesian approach to linkage analysis (eg, PPL), dense SNP linkage analysis (eg, EAGLET), generalized association analysis with both family-based and population-based components (eg, PPLD), and standard techniques in bioinformatics. As such, we extracted all sequence variants in the region, and computed the PPLD for the 9263 sequence variants observed in this family. To compare our methods to a commonly used filter-based approach (FBA) we:

1. Filtered out all common variants (ie, minor allele frequency ≥0.01) from the 1,215,400 total variants of chromosome 3.

2. Selected distant relatives (*n *= 3) over the age of 31 years with low SBP.

3. Filtered out variants that were not shared by the distant relatives in (2).

4. Used a phastCons 44-way threshold of 220 [8] to prioritize the variants that could not be filtered out by (1) and (3).

We performed the FBA analysis based on low SBP because the estimated trait model from the linkage analysis indicated that the locus would be protective against high SBP.

## Results

**Figure 1:**
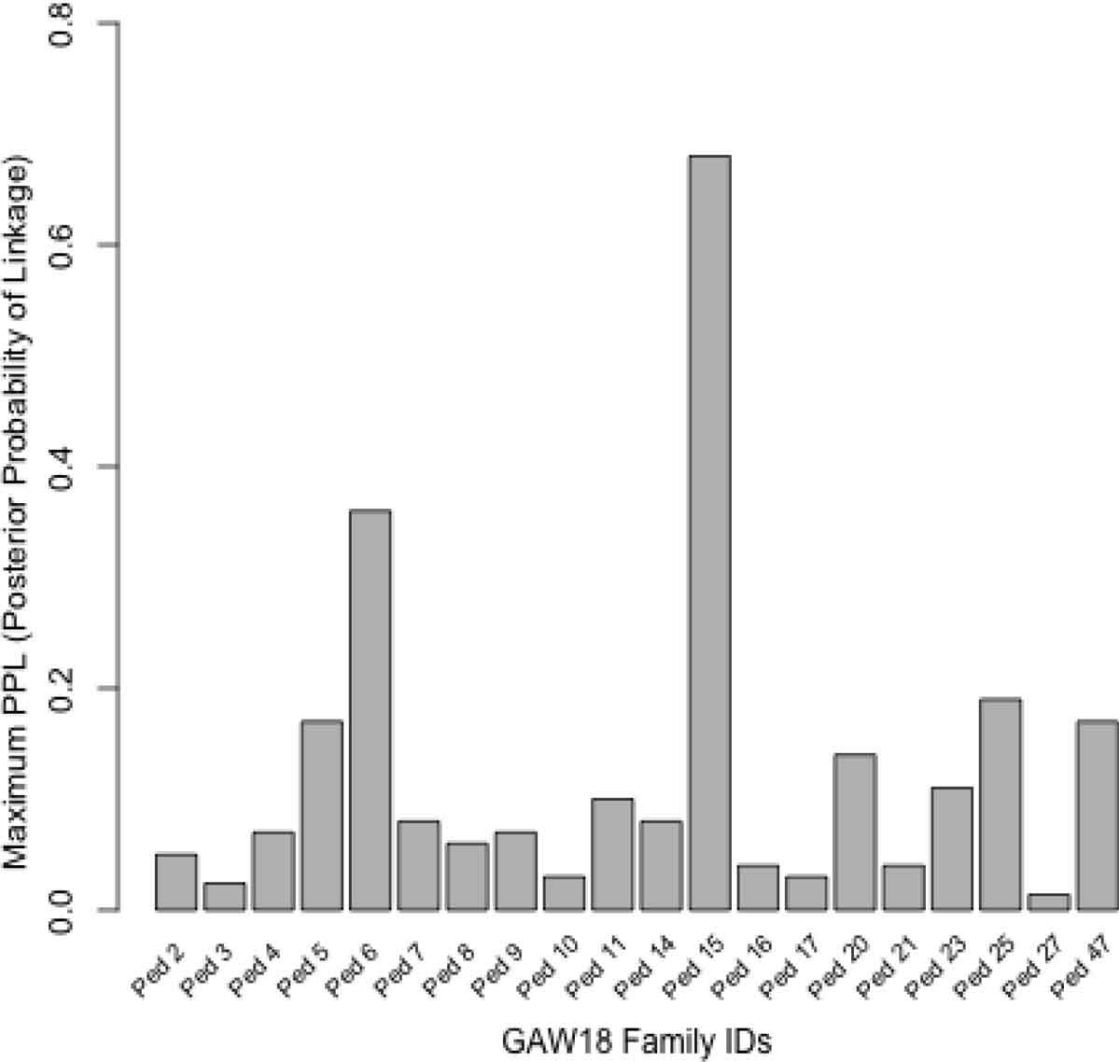
The maximum PPL is shown by family

**Figure 2:**
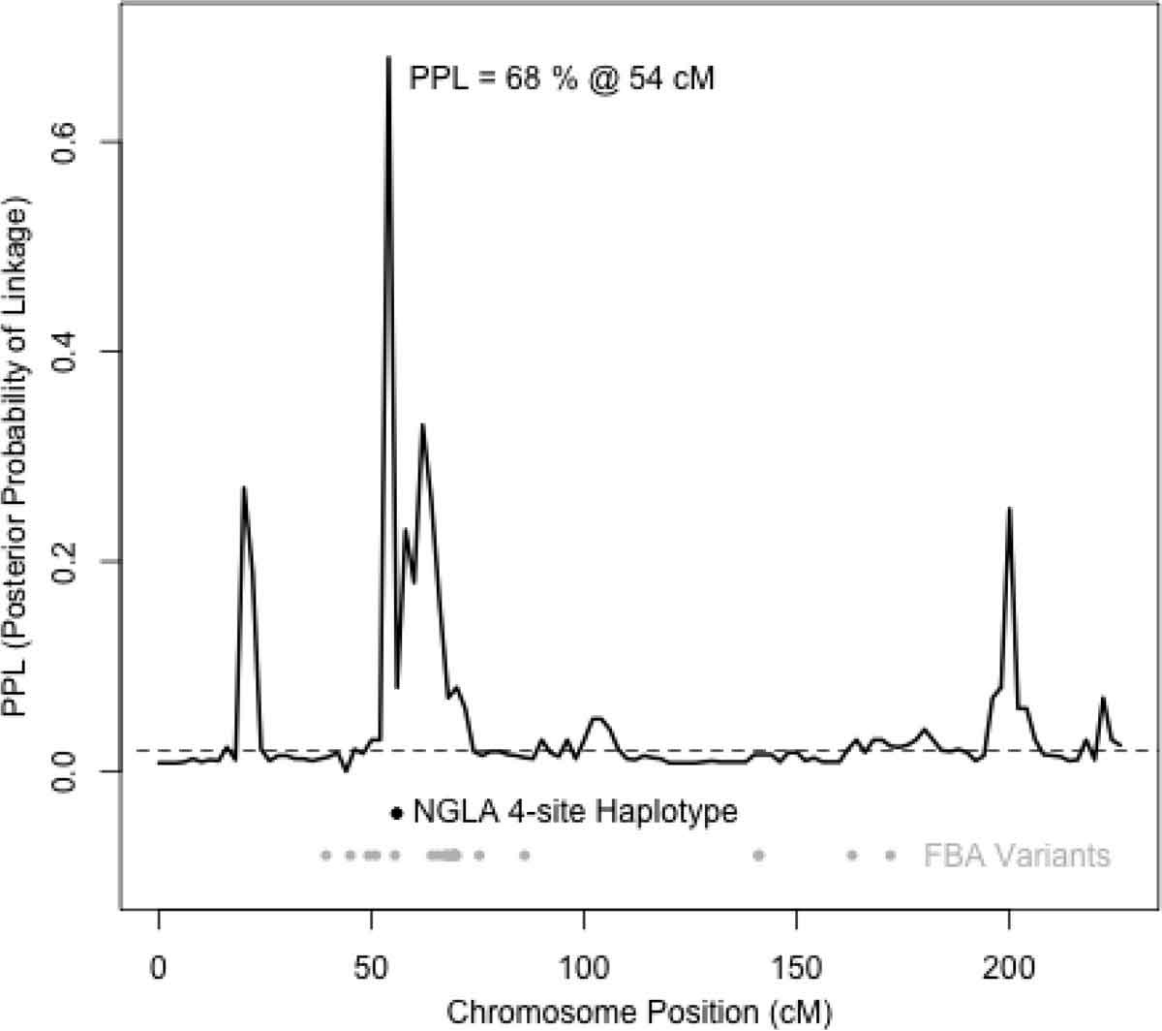
The PPL for Ped15 and sequence variants prioritized by NGLA (black dot) and FBA (gray dots). The dashed line is the prior probability for linkage

Of the 20 large Mexican American families, we focused on Ped15, which is the family with the strongest evidence for linkage (Figure 1; maxPPL = 68%; Bayes factor = 104.125). The linkage peak occurred at 54 centimorgans (cM) (Figure 2) and provided a narrow 4-cM region (52 cM, 56 cM) in which to follow-up with fine-mapping via the PPLD. In addition, we used EAGLET, LM_MARKERS, and a fixed trait model to compute the maximum of the average log of odds (LOD) score (2.43), where the average was taken over 15 random subsamples. Moreover, the 4-cM region identified by the PPL is completely contained within the EAGLET-based support interval (52.0, 62.9). The qualitative agreement between EAGLET, which is robust to the SNP selection procedure, and the PPL suggests that our PPL-based results are unlikely to be biased by the linkage map construction.

To fine-map the 4-cM region of interest, we applied the PPLD to all 9263 sequence variants. Note that in this region, 1059 of the GWAS SNPs are also present in the sequence data. The highest PPLD is 14% at variant 3_31907631, and the complete list of variants with PPLD scores greater than or equal to 4% is shown in Table 1. Note that the PPLD assumes a prior probability of LD = 0.04%, so that the seemingly low posterior probability of 4% is, in fact, a 100-fold increase over the prior probability of LD. Also recall that we are considering LD within a single family with just 18 founders. Variant 3_31590155 is located in a highly conserved intron (phastCons 44-way score = 294) of *OSBPL10*--a lipid receptor and potential candidate gene for high blood pressure [9]. In addition, this variant contributes to the rare 4-site haplotype (Table 1) that (a) only appears in families 4 and 15, and (b) lies directly beneath the PPL peak (see Figure 2). Also, note that family 4 provides some evidence for linkage at this locus (maximum PPL = 7%). Finally, to compare our NGLA methods to a commonly used FBA, we carried out the 4-step filtering procedure described in Background above. Similar to our NGLA methods, FBA identified variant 3_31590155. However, FBA also found 22 other variants spread across the entire chromosome (see Figure 2). Interestingly, rs7624739 (PPLD = 14%) was missed by FBA, and because it is not among the GWAS SNPs, it would also have been missed in a genome-wide association analysis.

**Table 1:** Characteristics of sequence variants identified by NGLA methods

Variant	Minor allele	MAF	PPLD	SNP	Intronic *OSBPL10*	Comment
3_30813267	G	0.3790	0.04	Y	N	
3_30833347	T	0.2115	0.04	Y	N	
3_30836121	A	0.2196	0.04	Y	N	Is also 1 of 65,519 GWAS SNPs
3_30836281	A	0.2169	0.04	Y	N	
3_30836986	T	0.2196	0.04	Y	N	
3_30837454	G	0.2246	0.04	Y	N	
3_30839168	G	0.2159	0.04	Y	N	
**3_31367917**	A	0.0017	0.04	N	N	Appears only in families 4 &15
**3_31590155**	A	0.0017	0.04	N	Y	Appears only in families 4 &15
**3_31688599**	A	0.0017	0.04	Y	Y	Appears only in families 4 &15
**3_31773725**	C	0.0017	0.04	N	Y	Appears only in families 4 &15
3_31907631	A	0.0729	0.14	Y	Y	AKA rs7624739

*MAF*, minor allele frequency.

Sequence variants 3_31367917,3_31590155,3_31688599, and 3_31773725 (in bold) form a rare 4-site haplotype that strongly segregates with low SBP.

## Discussion

It is important to remember that although our methodology-related results should be generalizable, our SBP-related results are primarily of a preliminary nature. Therefore, before any biological conclusions are drawn, the following caveats should be considered. First, the sequence data for Ped15 were entirely imputed. Ideally, the quality of that imputation--especially the existence and segregation of the 4-site haplotype--should be verified experimentally in the lab. Second, we did not adjust for measured covariates, which may have influenced some part (or all) of our results. Third, we confined our analyses to a 4-cM region of chromosome 3, which means that candidate genes in other regions (eg, MAP4 at 69 cM) cannot be excluded. As such, candidate genes in these regions deserve further investigation. Also, to the extent possible, we examined the sensitivity of our methods to MCMC error and to different phenotype definitions. In each case, our conclusions remained qualitatively the same.

Our NGLA approach to the analysis of sequence data led us to a manageable number of intronic (and intergenic) variants that lie in an excellent candidate gene: *OSBPL10*. Furthermore, the 4-site rare haplotype that we identified, was not seen in any of the unlinked families, but was seen in one of the other linked families. Although the commonly used FBA identified *OSBPL10*, it missed the strongest PPLD association: 3_31907631 (rs7624739), and it found several variants in apparently *unlinked *regions of chromosome 3 (PPL <2%).

## Conclusions

In summary, our methods have particularly nice features, including the posterior probability paradigm which is robust to trait model uncertainty; inherently ascertainment corrected; yields accurate localization given a dense marker map; returns a posterior probability rather than a *p *value; detects linkage (PPL) and association (PPLD); and, in this case, permits the simultaneous use of both quantitative (SBP) and dichotomous phenotypic information (ie, individuals affected with hypertension). Our approach also gives researchers the opportunity to make efficient use of all of the available dense SNP data, and permits the use any existing linkage analysis method that can handle multipoint analysis of dense SNP data in large pedigrees (eg, LM_MARKERS [7], SOLAR [10], etc). Therefore, if one restricts attention to the sequence data of a single large family, or if one studies multiple families, our NGLA methods provide excellent opportunities to map disease loci and to fine-map individual genes down to a handful of potentially pathogenic variants.

## Competing interests

The authors declare that they have no competing interests.

## Authors' contributions

VJV and DAG designed the overall study, WCLS and YH conducted statistical analyses and drafted the manuscript. All authors read and approved the final manuscript.
